# Hidden Markov Model: a shortest unique representative approach to detect the protein toxins, virulence factors and antibiotic resistance genes

**DOI:** 10.1186/s13104-021-05531-w

**Published:** 2021-03-30

**Authors:** Gary Xie, Jeanne M. Fair

**Affiliations:** Biosecurity & Public Health, Los Alamos National Laboratory, Mailstop M888, Los Alamos, NM 87545 USA

**Keywords:** Markov chains, Biological toxins, Virulence factors, Pathogenicity, Bacterial proteins, Genetic markers, High-throughput nucleotide sequencing, Microbiota, Open reading frames, Sensitivity and specificity

## Abstract

**Objective:**

Currently, next generation sequencing (NGS) is widely used to decode potential novel or variant pathogens both in emergent outbreaks and in routine clinical practice. However, the efficient identification of novel or diverged pathogenomic compositions remains a big challenge. It is especially true for short DNA sequence fragments from NGS, since sequence similarity searching is vulnerable to false negatives or false positives, as is mismatching or matching with unrelated proteins. Therefore, this study aimed to establish a bioinformatics approach that can generate unique motif sequences for profiling searching, resulting in high specificity and sensitivity.

**Results:**

In this study, we introduced a Shortest Unique Representative Hidden Markov Model (HMM) approach to identify bacterial toxin, virulence factor (VF), and antimicrobial resistance (AR) in short sequence reads. We first construct unique representative domain sequences of toxin genes, VFs, and ARs to avoid potential false positives, and then to use HMM models to accurately identify potential toxin, VF, and AR fragments. The benchmark shows this approach can achieve relatively high specificity and sensitivity if the appropriate cutoff value is applied. Our approach can be used to recognize the protein sequences of known toxins and pathogens, identifies their common characteristics and then searches for similar sequences in other organisms.

## Introduction

Several specialized databases (Tox-Prot [1], Virulence Factors Database [2], the Toxin and Virulence Database [3], Comprehensive Antibiotic Resistance Database (CARD) [4], and AR database (ARDB)) have been established to collect microbial toxin, virulence factor, and antimicrobial resistance genes sequences. One major limitation of these databases is that the VF and AR genes they contain are heavily biased towards easily cultivable model microbial organisms, making it difficult to identify remote homologues or novel resistance sequences present in fastidious or uncultured bacteria [5]. This bias complicates VF and AR gene identification across less commonly studied bacteria, a difficulty that is magnified by the diverse and complicated mechanisms of AR and VF. One potential solution to overcome this bias is to use the hidden Markov model (HMM) approach, which can find sequences with similar function but low sequence identity [6]. However, HMM-based approaches may have poor specificity and may not be able to distinguish between protein families with closely related functions.

One approach to mitigate false positive hits to regions of local homology is to identify and map against only unique substrings of protein sequences. Therefore, it is important to determine unique identifying strings ("markers") for all toxins, VF, and AR. For this reason, Curtis Huttenhower’s group first developed the ShortBRED (Short, Better Representative Extract Dataset) (https://huttenhower.sph.harvard.edu/shortbred) approach for assembly‐free identification of targeted genes in metagenomes [7]. ShortBRED builds short peptide sequences (markers) that are unique to a specific protein family, which are then used as a reference to map metagenomic reads. Since ShortBRED takes a set of protein sequences and reduces them to a set of unique identifying strings ("markers") for downstream Usearch [8], ShortBRED favors specificity (avoiding false positives) over perfect sensitivity (detecting all true positives). We recently further developed the Shortest Unique Representative Hidden Markov Model (SurHMM) approach to combine the best parts of ShortBRED and the hidden Markov model (HMM). Leveraging all existing ShortBRED markers, we are able to quickly and accurately assess the toxin, VF, and AR gene fragments presented among protein sequences of NGS reads/contigs or customer-ordered oligo nucleotide sequences by searching their specific motif-based profile.

## Main text

### Methods

#### Running SurHMM

**Fig. 1:**
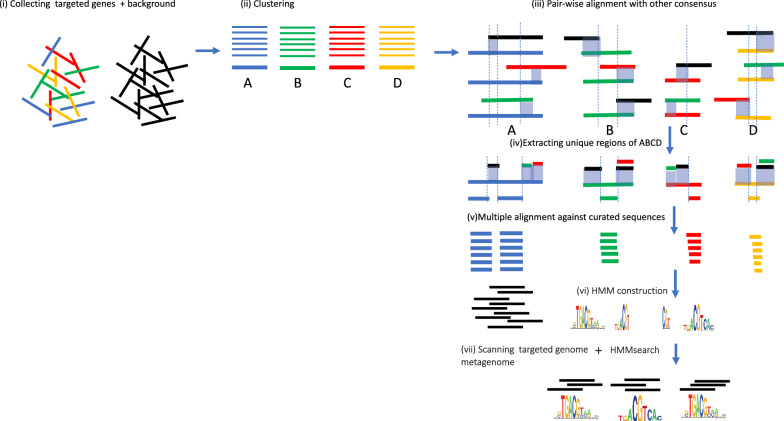
SurHMM approach creates Shortest Unique Representative Hidden Markov Model (SurHMM) for protein families of interest first, then identifies markers in targeted genomes and metagenomes by scanning predicted open reading frames or six-frame translation of given nucleotide reads. Drawing inspired from [7]

SurHMM has seven-step process shown in Fig. 1: (i) collecting protein sequences of interest (color) and background non-interest protein sequences from reference database (black), (ii) clustering protein of interest into families using CD-HIT [9], then generating consensus sequences (bold) of each family using MUSCLE alignment [10], (iii) using pair-wise sequence alignment to identify overlaps between consensus sequences and between consensus sequences and background non-interest proteins. Overlaps (blue shaded) represent non-unique peptides, (iv) extracting unique peptides by searching those regions that don’t overlap with any background references and other consensus, use them as unique representative markers, (v) taking marker sequences align with protein family of interest (from the step ii), (vi) constructs HMM profiles from alignment; (iv) and then scans the finished/drafted genome, or protein sequences from metagenomic reads/contigs using these HMM profiles to determine presence/absence of their corresponding families. SurHMM can be installed and run by following the instruction at GitHub (https://gitlab.com/gary_xie/surhmms). It consists of two parts: (1) ShortBRED-Identify that is identical to ShortBRED [7] covers steps (ii–iv)—This takes a FASTA file of amino acid sequences, searches for overlap among itself and against a separate reference file of amino acid sequences, and then produces a FASTA file of markers. (2) Hmmer-identify covers steps (v-vii)- This takes the FASTA file of markers and generates its corresponding HMM models, then can quantify their relative abundance in a protein FASTA file or identity its remote homologs in genome protein sequences.

#### Extracting pre-computed sequences of ShortBRED marker dataset

The consensus sequences of ShortBRED VF markers, an updated marker collection (mid-2017) for microbial virulence factors based on input protein sequences compiled from Victors [11], Virulence Factors Database (VFDB) [2], and MvirDB [12] were downloaded from bitbucket at the following site: https://bitbucket.org/biobakery/shortbred/downloads/ShortBRED_VF_2017_markers.faa.gz. Then a subset of bacterial toxins listed at the Centers for Disease Control Biological Select Agent and Toxin page were selected based on its metadata file, including Clostridium botulinum toxin, *Clostridium perfringens* Epsilon toxin, Staphylococcal enterotoxin B, Shiga-like toxin, and Vibrio cholerae toxin (https://emergency.cdc.gov/agent/agentlist-category.asp). The consensus sequences of corresponding toxin were extracted from the ShortBRED VF marker sequence file. Similarly, a subset of ShortBRED for markers of antibiotic resistance (AR) factors based on CARD [4] were also extracted from bitbucket at the following site: https://bitbucket.org/biobakery/shortbred/downloads/ShortBRED_CARD_2017_markers.faa.gz.

#### Generating a hidden Markov model

We generated HMM profiles for five toxin families, all VF families, and all AR families using HMMER (Eddy 1998) Version 3.1b2, February 2015. The multiple sequence alignment files (in STOCKHOLM format) were created for each marker by using the “phmmer” program search for each marker sequence against curated VFDB and CARD sequence databases, respectively. Then, profile HMMs were built with hmmbuild program (http://www.csb.yale.edu/userguides/seq/hmmer/docs/node19.html) for each toxin marker, as well as for all VF and AR gene markers. Toxin genes, VF, and AR gene profile databases were finally prepared using the hmmpress program, which can be found at http://manpages.ubuntu.com/manpages/bionic/man1/hmmpress.1.html. All curated hidden Markov models listed in Table 1 can be downloaded from GitHub (https://gitlab.com/gary_xie/surhmms).

**Table 1:** Summary of SurHMM generated in this study

Type	Profiles
Neurotoxin	61
Shiga_toxin	10
Choliz_toxin	8
Clostridium_perfringens toxin	21
Staphylococcal_toxin	74
Total virulence factors	86,136
Total antimicrobial resistance	3237

#### Positive control dataset

A set of 131 AR and 19 VF proteins were extracted from CARD [4] and VFDB [2] that were not used in training of the original profile HMMs, are treated as a true positive (TP). All corresponding toxin genes and all VF genes were extracted from VFDB [2] based on its metadata file. All AR genes from CARD [4] and ARDB [13] were extracted.

#### Negative control dataset

500 curated nonbacterial toxin protein sequences were downloaded from a supplementary site [14]. This set of data was extracted from Swiss-Prot [15] by combined search using the Sequence Retrieval System. The search was performed along with the "BUT NOT" option, using two information fields: (i) Comment with query word "function," and (ii) Comment using "toxin" as query word retrieved protein sequences that were examined manually in order to eliminate toxin proteins. This dataset is treated as a true negative (TN) in HMM validation test.

#### HMM validation

Hmmscan program (protein sequence vs profile-HMM database) from the HMMER 3.0 package (ftp://selab.janelia.org/pub/software/hmmer3/3.0/hmmer-3.0-linux-intel-x86_64.tar.gz), was used for searching non-toxin sequences (negative control), all downloaded VF genes (positive control) and AR genes (positive control) against toxin, VF, and AR HMM profiles respectively. Meanwhile, the hmmsearch program (profile-HMM vs protein sequence database) was used for searching toxin profiles against non-toxin protein sequences (-control). We set an E-value threshold (e value > 1e−5) for both hmmscan and hmmsearch programs.

### Results

We developed a method to quickly identify protein families of interest with high sensitivity by reducing protein families to short, unique, highly representative hidden Markov model (SurHMM) profiles (Fig. 1). To create these HMM profiles, our approach uses two inputs in step (i): (1) a FASTA file of proteins-of-interest and (2) a comprehensive reference database of background protein sequences. The protein sequences of interest were first clustered by global sequence homology to identify protein families, with each family collapsed to form a single consensus sequence in step (ii). Regions of a family’s consensus sequence that share strong, local sequence homology (“overlaps”) with proteins outside of the family of interest are then penalized. Based on these overlaps, short peptide markers of non-overlaps were isolated from the consensus that best represent the protein family. ShortBRED classifies these markers into three groups: *True Markers*, which do not overlap with the other protein families, *Junction Markers*, which overlap partially with the other protein families, and *Quasi Markers*, which are completely overlapped by another protein family. ShortBRED keeps those *True Markers and Junction Markers.* All Quasi Markers will be discarded. In the step (v), we took these consensus sequences of True Markers and Junction Markers aligned with curated proteins-family-of-interest from the step (ii), then followed by constructing hidden Markov models using hmmbuild command and hmmpress command to prepare an HMM profile database in the step (vi). The hmmscan command was used to search user-submitted protein sequences or oligonucleotide sequences (after six-frame translation) against the HMM profile database in the step (vii). The HMM creation process only needs to be run once for a given set of proteins, resulting in a reusable and distributable HMM profile database (Table 1). Creating a highly specific profile HMM database has three major advantages: (i) profile searches allow us to detect those remote homologues, which typically only have protein sequences conserved at some critical residues, (ii) searches against this profile database are more accurate, as the exclusion of non-specific (overlap) regions reduces false positive hits, and (iii) the HMM process is also very quick, as the search space is considerably reduced relative to the full database.

To evaluate SurHMM, we measured its accuracy in testing the known toxin genes and known non-toxin genes. For the positive control test, a subset of toxin gene and all VF genes were extracted from VFDB, as well as all AR genes were extracted from CARD. We tested the prediction accuracy of these 131 AR and 19 VF proteins not used in training of the original profile HMMs. These recruited protein sequences were subsequently incorporated into the corresponding AR and VF protein families, resulting in the final database of AR and VF SurHMM used for all further analyses in this study.

For the negative control test, a subset of non-toxin genes was extracted from Swiss-Prot. All default parameter settings were used, except the E-value threshold set at e value > 1e−5. All datasets performed as expected. SurHMM did not identify any false positive hits to non-toxin genes. In addition, SurHMM did increasing specificity by identifying remote homologues. For example. a toxin family in *Chryseobacterium piperi* with sequence similarity to botulinum neurotoxins [16] was identified using SurHMM (WP_034687877.1(1.8e−13), WP_034681281.1(2.5e−08), and WP_034687872.1 (7.9e−11)).

### Discussion

Other than ShortBRED and our SurHMM, several other online resources also provide VF and AR gene screening functions, such as VFanalyzer [2], PATRIC [17], VRprofile [18], and VirulenceFinder [19]. Many of them depend solely on BLAST searches. It is worth noting that in practical terms it is unrealistic to prevent the inclusion of bad BLAST hits in a typical large-scale data analysis since there is no universal cutoff to exclude bad BLAST hits from unrelated protein families. In general, any BLAST-like cutoffs are heuristic in nature; they are inevitably either too stringent or not stringent enough, so there is no perfect universal threshold that is suitable for all datasets. Due to these reasons and the inherent complexity of bacterial VF/AR, accurate in silico identification of VF and AR is still a challenging task. For example, although ShortBRED was able to keep false positives at a low level (< 5%), ShortBRED achieved true positive repeat and false positive repeat values comparable to or exceeding the centroids method, when increasing the number of protein families present and the share they comprised of the metagenome (S1 Table of [7]).

SurHMM can identify distant homologs through searching unique motifs that still have been preserved. Searches against SurHMM database are also more accurate, as the exclusion of non-specific (overlap) regions reduces false positive hits and the search space is considerably reduced relative to the full database. Therefore, SurHMM can obtain both higher speed and specificity relative to other approaches by reducing protein families to short, unique, highly representative hidden Markov model (SurHMM) profiles, In addition to identifying VF and AR genes, SurHMM approach can apply to other homology-based search for any protein family of interest, such as for novel pathogen detection, synthetic DNA screening, microbial communities profiling of protein families of interest, as well as aiding diagnosis or characterization of infections. Although HMM search can be used for identifying homologous protein or DNA sequences, our SurHMM profiled were trained by VF and AR protein sequences. Therefore, we can only scan protein sequences or six reading frame translations of DNA sequences against our SurHMM profiles. Theoretically, SurHMM approach could be applied to DNA sequence level, as well as eukaryotic sequences, if SurHMM profiles had been trained by appropriate datasets.

### Conclusion

Sequence similarity searching is vulnerable to false negatives or false positives, as mismatching or matching with unrelated proteins. Here, we are introducing the Shortest Unique Representative Hidden Markov Models (surHMM) approach for identifying potential bacterial toxin, virulence factor (VF), and antimicrobial resistance (AR) sequences. Since it combines the best parts of ShortBRED (Short better representative extract dataset) and the hidden Markov model (HMM), our approach generates unique motif sequences for profiling searching, resulting in high specificity and sensitivity.

## Limitations

In order to mitigate the lack of specificity and minimize false positives, SurHMM would need to use curated thresholds (for example, a gathering threshold) for each profile HMM. These profile-specific gathering threshold values would set an inclusion or exclusion bit score cutoff by comparing it with test datasets containing negative sequences.

## Data Availability

The protocol and precomputed HMMs are made available at https://gitlab.com/gary_xie/surhmms.

## Abbreviations

NGS: Next generation sequencing
HMM: Hidden Markov model (HMM)
VF: Virulence factor
AR: Antimicrobial resistance
ShortBRED: Short, Better Representative Extract Dataset
SurHMM: Shortest Unique Representative Hidden Markov Model

## References

[1] Jungo F, Bairoch A (2005). Tox-Prot, the toxin protein annotation program of the Swiss-Prot protein knowledgebase. Toxicon.

[2] Yang J (2005). VFDB: a reference database for bacterial virulence factors.. Nucleic Acids Res.

[3] Williams KP, Mantri Y (2004). Islander: a database of integrative islands in prokaryotic genomes, the associated integrases and their DNA site specificities.. Nucleic Acids Res.

[4] Raphenya AR (2016). CARD 2017: expansion and model-centric curation of the comprehensive antibiotic resistance database. Nucleic Acids Res.

[5] McArthur AG, Tsang KK (2017). Antimicrobial resistance surveillance in the genomic age. Ann N Y Acad Sci.

[6] Eddy SR (1998). Profile hidden Markov models. Bioinformatics.

[7] Kaminski J (2015). High-specificity targeted functional profiling in microbial communities with ShortBRED. PLoS Comput Biol.

[8] Edgar RC (2010). Search and clustering orders of magnitude faster than BLAST. Bioinformatics.

[9] Godzik A, Li W (2006). Cd-hit: a fast program for clustering and comparing large sets of protein or nucleotide sequences. Bioinformatics.

[10] Edgar R (2004). MUSCLE: a multiple sequence alignment method with reduced time and space complexity. BMC Bioinform.

[11] Zhao B (2018). Victors: a web-based knowledge base of virulence factors in human and animal pathogens. Nucleic Acids Res.

[12] Zhou, C.E., et al., *MvirDB—a microbial database of protein toxins, virulence factors and antibiotic resistance genes for bio-defence applications.* Nucleic Acids Research, 2006. **35**(suppl_1): p. D391-D394. 10.1093/nar/gkl791 PMC1669772 17090593

[13] Liu B, Pop M (2008). RDB—antibiotic resistance genes database. Nucleic Acids Res.

[14] Saha S, Raghava GP (2007). BTXpred: prediction of bacterial toxins. Silico Biol.

[15] Bairoch A, Apweiler R (2000). The SWISS-PROT protein sequence database and its supplement TrEMBL in 2000. Nucleic Acids Res.

[16] Mansfield MJ (2019). Bioinformatic discovery of a toxin family in *Chryseobacterium piperi* with sequence similarity to botulinum neurotoxins. Sci Rep.

[17] Warren A (2016). Improvements to PATRIC, the all-bacterial Bioinformatics Database and Analysis Resource Center. Nucleic Acids Res.

[18] Li J (2017). VRprofile: gene-cluster-detection-based profiling of virulence and antibiotic resistance traits encoded within genome sequences of pathogenic bacteria. Brief Bioinform.

[19] Joensen KG (2014). Real-time whole-genome sequencing for routine typing, surveillance, and outbreak detection of verotoxigenic *Escherichia coli*. J Clin Microbiol.

